# The Spirit of Charity and Its Origin

**Published:** 1921-06-11

**Authors:** 


### THE HISTORY OF MEDICINE.

### The Spirit of Charity and its Origin.

The history of medicine affords us not only a subject of study to help us in our practice, but also one of literary interest to delight us in our lighter moments. It is those parts of the subject nearest to our own times which are of most use to us in our daily work, and the lives of Hunter, of Pasteur, and of Paget will for many years afford subjects meet to be read by all who practise medicine. But that a study of a longer period may afford true practical value is shown by the paper read before the Historical Section of the Royal Society of Medicine by Dr. Raymond Crawford on typhus and the bite of the louse in 1912; while the same writer's "Plague and Pestilence in Literature and Art" is the best example of the other aspect of the subject.

### Mediæval Medicine.

Under the general editorship of Dr. John D. Comrie, Messrs. A. and C. Black have published a series of medical history manuals, of which the volume on "Mediæval Medicine," by Dr. James J. Walsh, of New York, is before us. Dr. James Walsh is an enthusiastic admirer of the Middle Ages, which he defines as lasting from 476 A.D. to the fall of Constantinople in 1453; but while we are willing to agree with him that the term "Dark Ages" contains too opprobrious an epithet for this period, we do not feel that he has persuaded us fully to share his enthusiasm. Dr. Walsh wishes us to believe that this excellence of mediæval medicine was entirely due to Greek influence. To do this he belittles Rome, and infers that the Romans were mere barbarians, who swept away a former civilisation both in Etruria and in Carthage. In reading about these wars our sympathies have always been with the Carthaginians, and with Hannibal in particular, but our reason has told us that it was good for Western civilisation that it was Rome, and not Carthage, which was the conqueror, for it gave the command of the Western Mediterranean to a race eminently suited to absorb knowledge, customs, and culture from all with whom they came in contact. And if the Romans could absorb as much from Greece as they did, we cannot imagine that they took nothing from those other races whom they conquered before ever they met the Greeks, nor that they added nothing new, whether constructive or critical, to that which they absorbed. In considering the lines of descent of our modern medicine, Dr. Walsh seems to us to have done a similar injustice to the Arabs, who were, he infers, merely a vehicle to convey Greek culture down to our times. We know that when Islam gave a stimulus to the Arabs and started them on their career of conquest, ultimately to extend from the pillars of Hercules to the Persian Gulf, they had no art and no culture of their own, and we know they employed Greeks to build for them and to teach them, but we cannot believe that Greek culture was their only source of art and knowledge, nor necessarily that it was their greatest. At Baghdad they came in touch with Persia, and through that had intercourse with India. At Cairo they employed Copts in the same way as farther north they employed the Greeks. The visitor to Jerusalem sees in the Dome of the Rock the most perfect specimen of Byzantine art, but the visitor to the hanging Coptic church recognises that here can be seen already developed much of what his study of the mosques in Cairo has made him imagine is most typical of Arab art. We have given one example from a sister art, because in it the deeds and thoughts of a thousand years ago are to be seen to-day, but we have no reason to doubt that when they absorbed ideas of the art of architecture they also absorbed ideas of the art of medicine, and the Copts had inherited from the Egyptians a medicine in the same way as the Byzantine Greeks did from the Athenians. So also we may infer they received ideas from Judean thought and from Judaism, upon which their religion was largely based; nor can we doubt that they profoundly modified everything that came into their hands. In the decorative arts their powers of development were fettered by the rigid monotheism of their creed, which forbade them to make a graven image of any living thing; but in the realm of thought they were not so limited, and they brought to the problems of life that depth of contemplation which their country inspires. At Karnak by night the tourist acquires some glimmering of this, but the visitor to Karnak is among the ruins of temples made by man, and returns from his reverie to his Winter Palace Hotel with its coffee and its cocktails. When the traveller in the desert or the steppe pitches his bivouac the beauty of the night leads him to refrain from sleep, while the depth of the sky and the brightness of the stars cause him slowly to revolve in his mind the subjects of life and death, of eternity of time and space, and of the relations of these to human beings. If a short sojourn in the desert has this effect upon the hustler from the West, how much more must a hereditary existence of thousands of years inspire the whole life and thought of those who come thence!

### The Hospital Spirit.

There is another subject in which we believe that Dr. Walsh takes too narrow a view. It is raised in those passages of his book from which we gather that he looks upon the hospital spirit as being purely Christian in origin. The Muristan of Kalaun in Cairo bears present-day witness to the same spirit in the sister religion founded by Mahomet, and the hospitals in every capital founded and maintained by Jews bear witness to the same spirit in modern Judaism. It is true that both these may have been stimulated to emulation by the sight of hospitals springing up in Christian countries, yet the germ of idea in all three may be traced back again to the school of thought in Judaism which developed in Alexandria shortly after the founding of that city, and which is known to the modern reader through the sayings of Jesus the Son of Sirach, when he says, "Reject not the supplication of the afflicted," or "Be not slow to visit the sick, for that shall make thee to be beloved."

But before Herod ruled in Jewry hospitals had arisen in India under the spirit of Buddhism, and when the Spaniards conquered the New World they found such institutions in one at least of the races there. Neither of these can have received the idea from Christianity, and serve to show that the spirit of charity rose separately in different races as man's mind strove towards civilisation. There is no doubt that it has up to now attained its greatest growth in those countries inherited by Christian races, and we believe that the tenets of the creed to which the people clung afforded it that stimulus which caused it to grow; but we cannot think that it makes this progress any greater or more worthy by denying its parallel growth amongst other civilisations, and we are confident that the founder of Christianity would not have subscribed to such a view.

We have been at some pains to disagree with certain aspects of the argument in an illuminating book, because we believe that they involve principles which are contrary to the best spirit of medical education and of the teaching of the day. This is that spirit which tells us there is no place and no person, no work and no thought, however small or poor, however unassuming or modest, whence we may not learn. If we imagine that mediæval medicine owed all to the Greeks we shall sit at the feet of one master and listen only to what he says. If we think that the spirit of charity arose from the thoughts of one only of the teachers of mankind, we shall lose the value of a wide outlook on human nature. We must go out into all the world teaching what we know and by our teaching learn from everyone we meet.

